# A LIVE ONLINE EXERCISE INTERVENTION FOR OLDER ADULTS: A MIXED METHODS RANDOMIZED CONTROLLED TRIAL

**DOI:** 10.1093/geroni/igad104.1977

**Published:** 2023-12-21

**Authors:** Giulia Coletta, Kenneth Noguchi, Kayla Beaudoin, Angelica McQuarrie, Meridith Griffin, Ada Tang, Stuart Phillips

**Affiliations:** McMaster University, Hamilton, Ontario, Canada; McMaster University, Hamilton, Ontario, Canada; McMaster University, Hamilton, Ontario, Canada; McMaster University, Hamilton, Ontario, Canada; McMaster University, Hamilton, Ontario, Canada; McMaster University, Hamilton, Ontario, Canada; McMaster University, Hamilton, Ontario, Canada

## Abstract

Regular physical activity (PA) and exercise alleviate mental health and age-related physical declines in older adults; however, strategies are needed to help older adults engage in PA and exercise. Live online video may be a viable mode of delivery for exercise programs. Thirty-one sedentary community-dwelling older adults (65-80 years) were randomized to an 8-week live online exercise program (ACTIVE, n=16; age 70±4 years; 69% women) or waitlist control (CON, n=15; age 72±5 years; 87% women) group. Quantitative effectiveness data were collected pre-and post-intervention and included accelerometry-derived PA levels and mental health (depression, anxiety, and loneliness). Feasibility outcomes included attendance and participant satisfaction. Semi-structured interviews were optional for all participants after completing the online exercise program (n=22; ACTIVE=12; CON=10; 73% women). The ACTIVE group attended 97% of the online classes, and 98% reported satisfaction with the program. Post-intervention, there were no differences between groups on daily steps (ACTIVE=4336 ± 2638 steps/d; CON=3125 ± 1250 steps/d), daily activity energy expenditure (ACTIVE=227 ± 249 kcal/d; CON=119 ± 166 kcal/d), and moderate-to-vigorous physical activity (ACTIVE=42 ± 44 min/d; CON=23 ± 30 min/d). An effect of the intervention was observed in the ACTIVE group for symptoms of depression (3.9±2.4 to 2.0±1.7; p=0.015). There was no intervention effect compared to CON on anxiety or loneliness. Common themes identified from qualitative interviews included satisfaction with the program and perceived health improvements. A live online exercise program was a feasible approach to delivering physical activity to older adults.

